# MadureseSet: Madurese-Indonesian Dataset

**DOI:** 10.1016/j.dib.2023.109035

**Published:** 2023-03-07

**Authors:** Noor Ifada, Fika Hastarita Rachman, M Wildan Mubarok Asy Syauqy, Sri Wahyuni, Adrian Pawitra

**Affiliations:** a Informatics Department, Engineering Faculty, University of Trunojoyo Madura, Bangkalan 69162, Indonesia; b Mechatronics Department, Engineering Faculty, University of Trunojoyo Madura, Bangkalan 69162, Indonesia; c Yayasan Pragalba, Bangkalan, 69162, Indonesia

**Keywords:** Database, Dictionary, Madura, Indonesia, NLP

## Abstract

MadureseSet is a digitized version of the physical document of Kamus Lengkap Bahasa Madura-Indonesia (The Complete Dictionary of Madurese-Indonesian). It stores the list of lemmata in Madurese, i.e., 17809 basic lemmata and 53722 substitution lemmata, and their translation in Indonesian. The details of each lemma may include its pronunciation, part of speech, synonym and homonym relations, speech level, dialect, and loanword. The framework of dataset creation consists of three stages. First, the data extraction stage processes the scanned results of the physical document to produce corrected data in a text file. Second, the data structural review stage processes the text file in terms of the paragraph, homonym, synonym, linguistic, poem, short poem, proverb, and metaphor structures to create the data structure that best represents the information in the dictionary. Finally, the database construction stage builds the physical data model and populates the MadureseSet database. MadureseSet is validated by a Madurese language expert who is also the author of the physical document source of this dataset. Thus, this dataset can be a primary source for Natural Language Processing (NLP) research, especially for the Madurese language.

**Specifications Table**

**Table:** 

Subject	Computer Science
Specific subject area	Madurese corpus, Madurese Natural Language Processing, Madurese-Indonesian Machine Translation
Type of data	Table, MySQL database
How the data were acquired	The dataset was collected from a physical document of Kamus Lengkap Bahasa Madura-Indonesia [1]. The document was scanned using a scanner machine as a PDF file. The PDF file was then optimized using an open-source PDF manipulation tool k2pdfopt (https://www.willus.com/k2pdfopt/), PDF Optical Character Recognition (OCR) was conducted using Adobe Acrobat software, PDF Conversion was performed using the “pdftotext” Python package (https://pypi.org/project/pdftotext/). We subsequently used Python programming and text editor software to convert the text to paragraphs and manual correction. The MySQL database was populated using Python based on the structural review process.
Data format	Raw, Analyzed, Filtered, Processed
Description of data collection	The dataset is collected and processed in three stages: First, the physical dictionary document is scanned, optimized, recognized by Optical Character Recognition (OCR), converted to text, and manually corrected. Second, the text goes through a semi-automatic review process where a human expert manually reviews and analyzes the data structure that best represents the information in the dictionary to generate the rules for automatic data processing. The data structure includes the paragraph, homonym, synonym, linguistic, poem, short poem, proverb, and metaphor structures. Last is the database construction stage, where we build the physical data model and populate the data for the database.
Data source location	Indonesia
Data accessibility	Repository name: Mendeley Data Direct URL to data: https://data.mendeley.com/datasets/nvc3rsf53b/1 DOI: 10.17632/nvc3rsf53b.1
Related research article	-

## Value of the Data

• MadureseSet is a Madurese-Indonesian dictionary database that includes the list of lemmata as well as the descriptions of part of speech (POS), loanwords, and speech levels
• It can be used as a primary source to support research in Natural Language Processing (NLP) specifically for Madurese language, such as:
  ○ Madurese stemming
  ○ Madurese POS tagging
  ○ Madurese word sense disambiguation
  ○ Madurese spelling correction checker
  ○ Madurese machine translation for different language speech level
  ○ Madurese-Indonesian machine translation
  ○ MadureseSet can also be used to support research in the education sector, i.e., the development of an educational learning application for Madurese
• The dataset is validated by a Madurese language expert who is also the author of the source of this dataset, i.e., Complete Dictionary of Madurese-Indonesian [1]

## 1 Objective

Madurese is ranked third out of ten of Indonesia's most spoken regional languages [2] and is in fifth place out of ten of Indonesia's most populous ethnic groups [3]. The Madurese language's technological development is limited to a lexical dictionary available in web-based [4] and Android-based [5] applications. Developing linguistic-based applications such as Natural Language Processing (NLP) and education learning requires an intensive analysis of the word-by-word relationship and the Madurese-Indonesian dataset. In this case, the mere implementation of lexical-based applications is insufficient. The study on the Madurese language is ongoing. Its detailed supporting component still requires thorough work, especially since the Madurese-Indonesian language dataset that provides the part of speech description and word relations is yet to be available. The Madurese-Indonesian dataset is a primary resource in the Madurese language study. In other words, its non-existence makes it more challenging to research Madurese language (structural writing and translation), and improper implementation of components on NLP applications can impact their performance quality.

## 2 Data Description

**Fig. 1:**
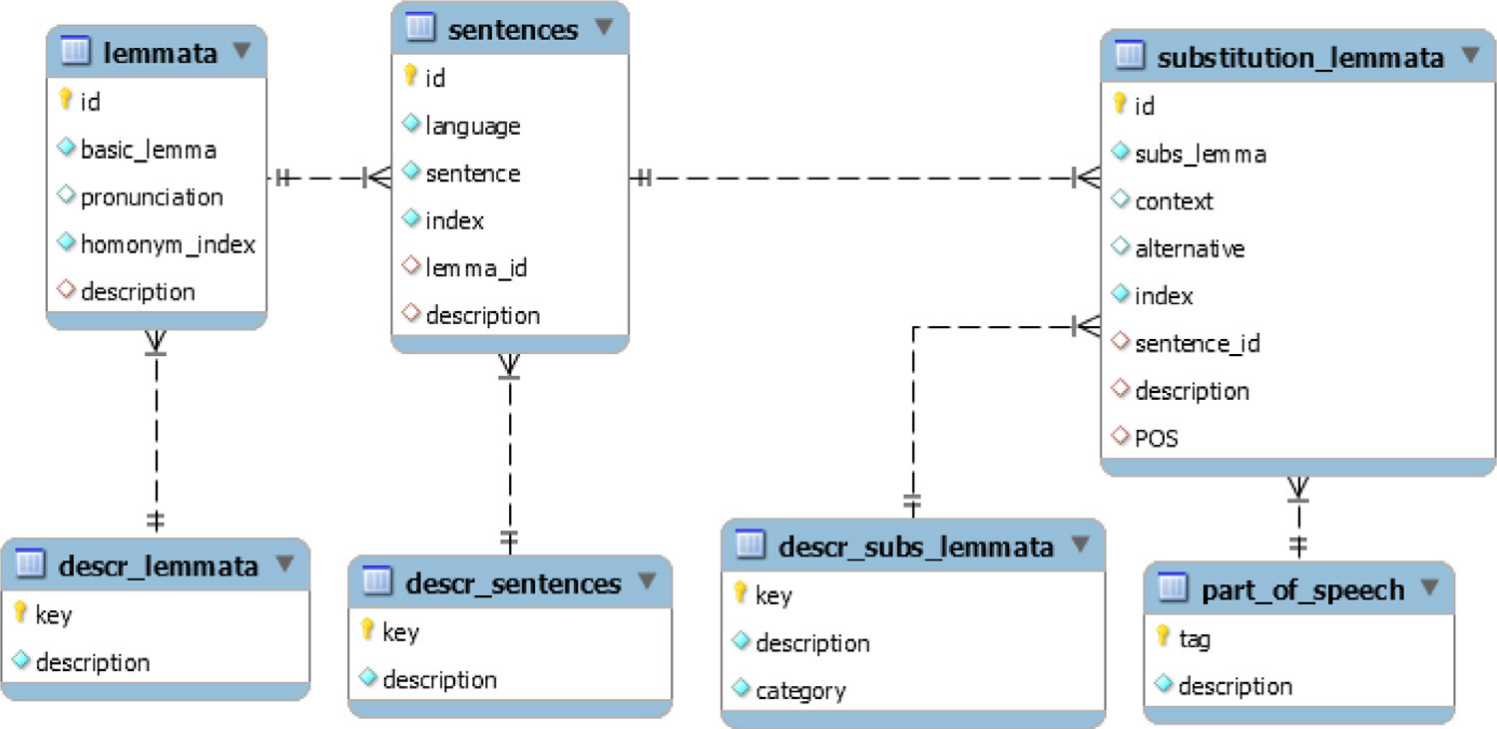
Madureseset database physical data model.

**Fig. 2:**
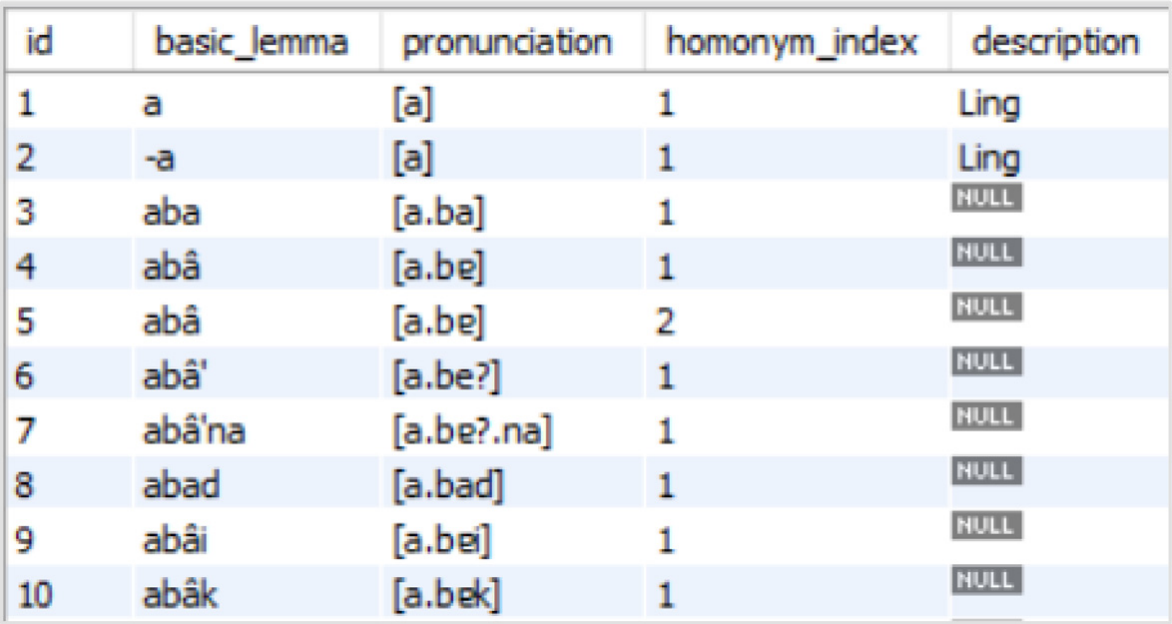
Screenshot of data in “lemmata” table.

**Fig. 3:**
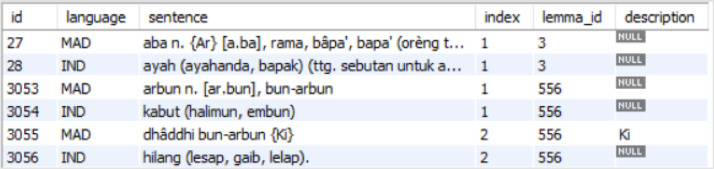
Screenshot of data in “sentences” table.

**Fig. 4:**
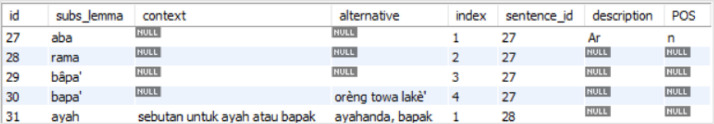
Screenshot of data in “substitution_lemmata” data.

MadureseSet is a Madurese-Indonesian dictionary database built using MySQL database software. Fig. 1 shows the database model where we can observe the seven different tables and the relations amongst tables in the database. The database stores a list of lemmata, pronunciation, descriptions of linguistics, part of speech (POS), loanwords, dialects, and speech levels. Table 1 presents the detail of each table. The main tables of MadureseSet that store the dictionary data are the “lemmata”, “sentences”, and “substitution_lemmata” tables. At the same time, the other four tables are for storing the descriptions related to the main tables. Figs. 2, 3, and 4 show screenshots of the data in the three tables. We can further find five detailed pieces of information from the Madurese-Indonesian dictionary based on the relations among all tables. **First**, we can get the statistics of pronunciation, homonym, and linguistic description of 17809 lemmata based on the relationship between the “lemmata” and “descr_lemmata” tables (Table 2). **Second**, we can produce the form of literature description of the 36086 Madurese and 36086 Indonesian sentences based on the relationships between the “lemmata”, “sentences”, and “descr_sentences” tables (Table 3). The description includes proverb, poem, short poem, and metaphor. **Third**, the relationship between “sentences” and “substitution_lemmata” tables defines the statistics of synonyms of 53772 Madurese and 39934 Indonesian substitution lemmata (Table 4). **Fourth**, the relationship between the “substitution_lemmata” and “part_of_speech” tables outline the statistics of part of speech of the substitution lemmata (Table 5). **Last**, details of the loanwords, dialect, and speech level are derived based on the relationship between the “substitution_lemmata” and “descr_sub_lemmata” tables (Table 6).

**Table 1:** Description of tables of madureseset database.

Name of Table	Description
lemmata	List of lemmata and their pronunciation, homonym index, and description. The description in this table refers to the primary key in the “descr_lemmata” table
descr_lemmata	List of lemmata description in terms of affixation or linguistic label
sentences	List of sentences in Madurese, the Indonesian translation, and description of each lemma. The lemma in this table refers to the primary key in the “lemmata table”. Meanwhile, the description refers to the primary key in the “descr_sentences” table
descr_sentences	List of sentences description based on four Madurese forms of literature, i.e., Proverb, Poem, Short Poem, and Metaphor
substitution_lemmata	List of substitution lemmata, context, alternative, part of speech, and the description of each sentence. The sentence refers to the primary key in the “sentences” table”. The part of speech and description subsequently refer to the primary keys in the “part_of_speech” and the “descr_subs_lemmata” tables
descr_subs_lemmata	List of various substitution lemmata descriptions that consist of:
	• 6 (six) dialects: Bangkalan, Bangkalan & Pamekasan, Bangkalan & Sumenep, Pamekasan, Pamekasan & Sumenep, and Sumenep
	• 4 (four) speech levels: Alos Tengghi, Alos, Tengngaan, and Lomra
	• 2 (two) types of loanwords:
	○ 11 (eleven) foreigns: Arabic, Dutch, China, Family, Indonesian, English, Latin, Portuguese, French, Persian, and Tamil
	○ 2 (two) locals: Javanese and Sanskrit
part_of_speech	List of 10 (ten) parts of speech: Adjective, Adverb, Noun, Numeral, Particle, Pronoun, Personal pronoun_first person singular, Personal pronoun_first person plural, Personal pronoun singular, and Verb

**Table 2:** Counts based on the relationship between “lemmata” and “descr_lemmata” tables.

Attribute		Number of data
Lemma		17809
Pronunciation		15620
Homonym (pairs of lemmata that have the exact spelling)	All Pairs	4037
	Two pairs	2968
	Three pairs	822
	Four pairs	152
	Five pairs	70
	Six pairs	18
	Seven pairs	7
Description	Linguistic	15

**Table 3:** Counts based on the relationship between “lemmata”, “sentences”, and “descr_sentences” tables.

Attribute		Number of data	
		Madurese	Indonesian
Lemma		17809	-
Sentence		36086	36086
Sentence Description	All description	1812	-
	Metaphor	593	-
	Short Poem	32	-
	Proverb	1116	-
	Poem	71	-

**Table 4:** Counts based on the relationship between “sentences” and “substitution_lemmata” tables.

	Attribute	Number of data	
		Madurese	Indonesian
Sentence		36086	36086
Substitution Lemma		53772	39934
Synonym (pairs of substitution lemmata that are in the same sentence)	All Pairs	29128	6830
	Two pairs	15228	4752
	Three pairs	7173	1227
	Four pairs	3564	580
	Five pairs	1570	200
	Six pairs	774	42
	Seven pairs	378	21
	Eight pairs	264	8
	Nine pairs	72	0
	Ten pairs	10	0
	Eleven pairs	22	0
	Twelve pairs	60	0
	Thirteen pairs	13	0

**Table 5:** Counts based on the relationship between “substitution_lemmata” and “part_of_speech” tables.

Attribute		Number of data	
		Madurese	Indonesian
Substitution Lemma		53772	39934
Substitution Lemma Description	All Parts of Speech	25590	-
	Adjective	3750	-
	Adverb	296	-
	Noun	9206	-
	Numeral	160	-
	Particle	236	-
	Pronoun	150	-
	Personal Pronoun Singular	1	-
	Personal Pronoun Plural	1	-
	First personal pronoun	21	-
	Verb	11769	-

**Table 6:** Counts based on the relationship between “substitution_lemmata” and “descr_sub_lemmata” tables.

Attribute			Number of data	
			Madurese	Indonesian
Substitution Lemma			53772	39934
Loanword Description	All Loanwords		768	1051
	Foreign	Arabic	364	0
		Dutch	153	0
		Chinese	15	0
		Family	0	22
		Indonesian	66	0
		English	27	1
		Latin	0	933
		Portuguese	24	0
		French	1	3
		Persian	5	0
		Tamil	3	0
	Local	Javanese	90	92
		Sanskrit	20	0
Dialect Description	All Dialects	214	-	
	Bangkalan	127	-	
	Bangkalan and Pamekasan	5	-	
	Bangkalan and Sumenep	1	-	
	Pamekasan	33	-	
	Pamekasan and Sumenep	12	-	
	Sumenep	36	-	
Speech Level Description	All Speech Levels	2672	-	
	Alos Tèngghi	676	-	
	Alos	605	-	
	Tengngaan	242	-	
	Lomra	1149	-	

## 3 Experimental Design, Materials and Methods

**Fig. 5:**
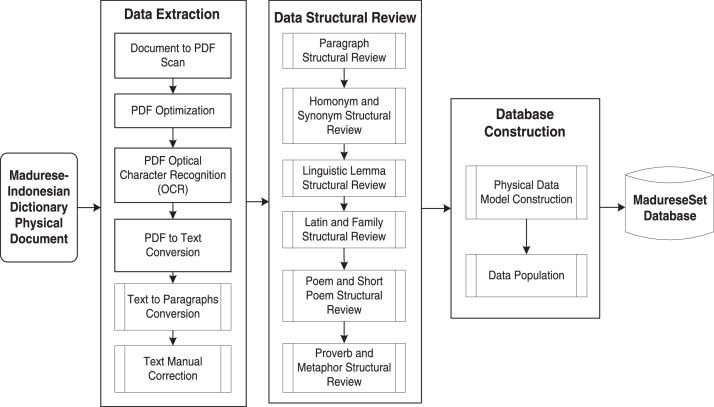
Dataset creation framework.

The Madurese dataset created in this work was entirely collected from The Complete Dictionary of Madurese-Indonesian (Kamus Lengkap Bahasa Madura-Indonesia) [1]. The framework of dataset creation consists of three stages, as illustrated in Fig. 5.

## 4 Data Extraction

**Fig. 6:**
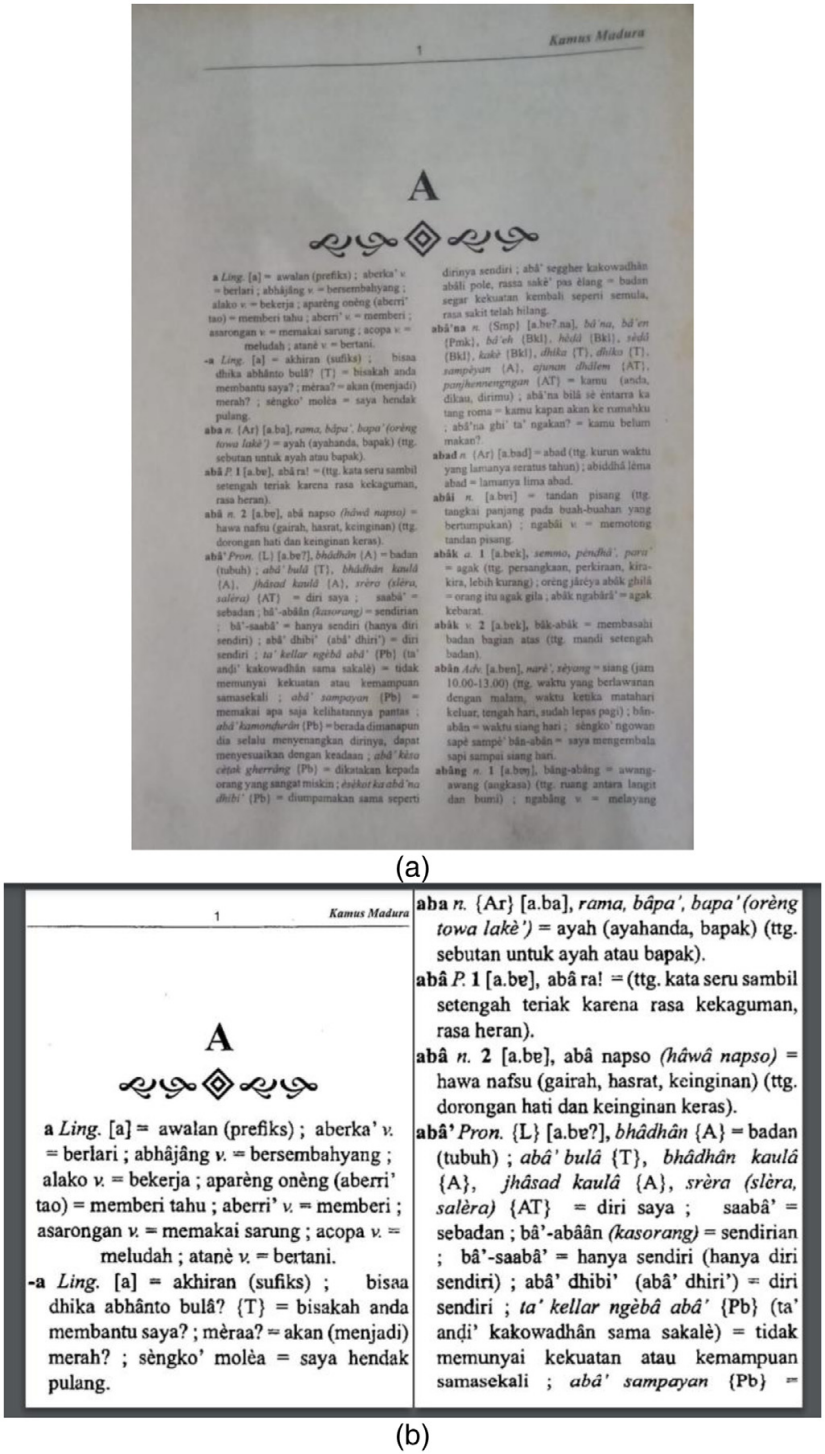
Example of comparison of PDF results from: (a) Scan and (b) Optimization processes.

**Fig. 7:**
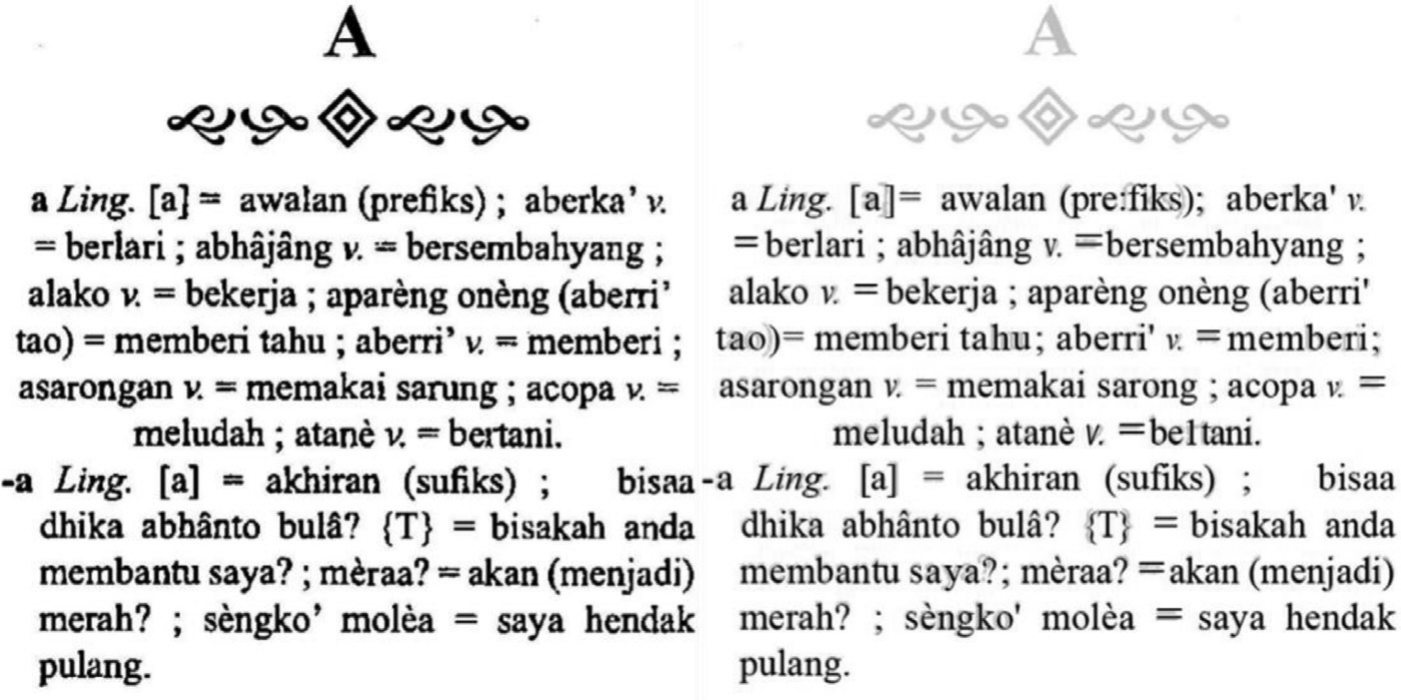
Example of PDF result from the OCR process.

**Fig. 8:**
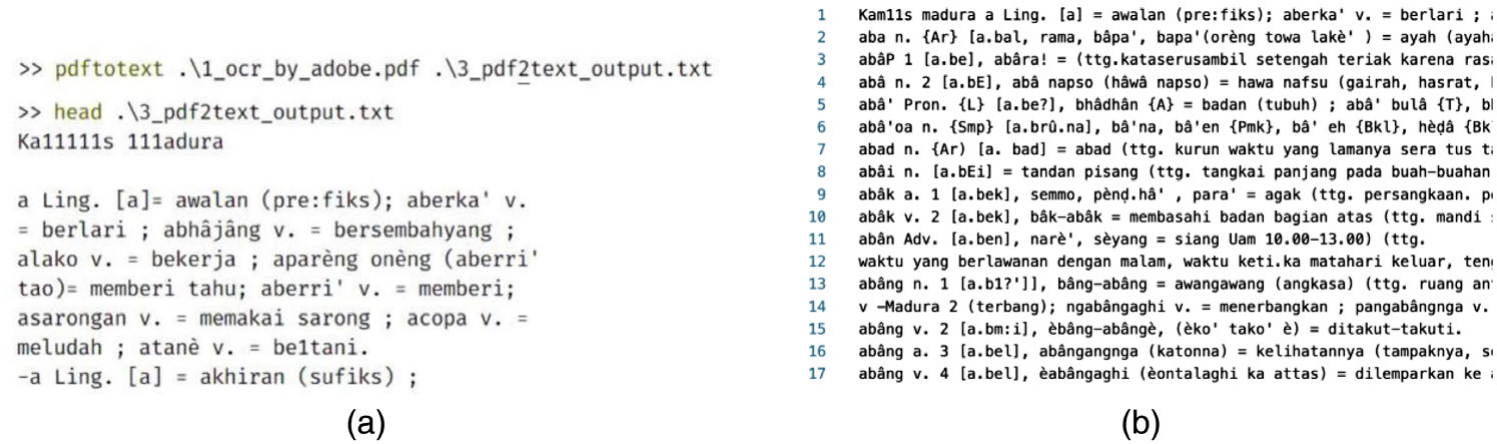
Example of comparison of results: (a) PDF to text conversion and (b) Text to paragraph conversion processes.

The data extraction stage consists of six processes. **First**, we scan and convert the physical document to PDF using a 600dpi optical resolution scanner. This process results in a PDF file containing 738 pages of images of texts. **Second**, the PDF optimization process optimizes the PDF file using the k2pdfopt tool (https://www.willus.com/k2pdfopt/). This process minimizes the errors of subsequent Optical Character Recognition (OCR) process, resulting in an optimized PDF file containing 3800 pages of images of texts. **Third**, PDF OCR converts the optimized PDF from images of texts into a machine-readable text format using Adobe Acrobat software. We set the language setting recognition to French to recognize the unique characters of Madurese. As a result, OCR can recognize the â and è characters, yet it fails to detect the “ḍ” and “ṭ” characters and therefore require manual correction. Fig. 6 shows an example of a comparison of PDF results from the scan and optimization processes. While Fig. 7 shows an example of a PDF result from the OCR process. **Fourth**, PDF-to-text conversion is the process where the result of the PDF OCR file is converted to a text file using the “pdftotext” Python package (https://pypi.org/project/pdftotext/). **Fifth**, using Python programming, text-to-paragraph conversion correctly ensures the representation of each lemma into distinct paragraphs. Fig. 8 shows an example of comparing text files resulting from PDF-to-text conversion and text-to-paragraph conversion processes. The **final process** is manual correction, i.e., manually fixing errors. Examples of OCR errors are the misinterpretations of “ḍ” and “ṭ” characters, missing signs (period “.” at the end of a lemma, equal “=”), incorrect usage of the semicolon sign “;”, missing pair of bracket/parenthesis/square bracket, inconsistencies in part of speech labeling, and inconsistencies in dialect labeling. We can fix some errors by using the “Find and Replace” feature using Text Editor software.

## 5 Data Structural Review

The data structural review stage aims to achieve the data structure that best represents the information in the dictionary and consists of six processes. We conduct this stage semi-automatically, where a human expert manually reviews and analyzes the data structure to generate the rules for automatic data processing. **First**, the paragraph structural review examines the pattern of a lemma. Each Madurese lemma has part of speech, pronunciation (located within the square brackets sign “[]”), Indonesian translation (found after an equal sign “=”), and examples of sentences (separated using semicolon sign “;”). A lemma can also have three translation text categorizations, i.e., the standard and alternative translations located within the parentheses sign “()”, and the context translation located within the parentheses sign “()” started with “ttg.” label. **Second** is the structural review of homonyms and synonyms. A homonym is a condition when two or more words have the exact spelling or pronunciation but different meanings. A synonym is a condition when a word has the same meaning as another in the same language. We recognize a homonym if a lemma has a number label right after it, while a synonym is labeled and separated using the comma sign “,”.The **third** is the linguistic structural review. Some lemmas are labeled as linguistic (“Ling.”), meaning they are affixes in Madurese. **Fourth** is the Latin and Family structural reviews. Latin (labeled as “{Lt}”) and Family (labeled as “{Fam}”) are the translation of animal or plant species. **Fifth** is the poem and short poem structural reviews. A poem (labeled as “{Ptn}”) and a short poem (labeled as “{Krm}”) are a chain of words; thus, we disregard the regular usage of the comma “,” for synonym labeling. The **final process** is the proverb and metaphor structural reviews. Proverbs (labeled as “{Pb}”) and Metaphors (labeled as “{Ki}”) have implicit meaning, and therefore they are not directly translated. The location of the explanation or description of the text is within parentheses “()”.

## 6 Database Construction

**Fig. 9:**
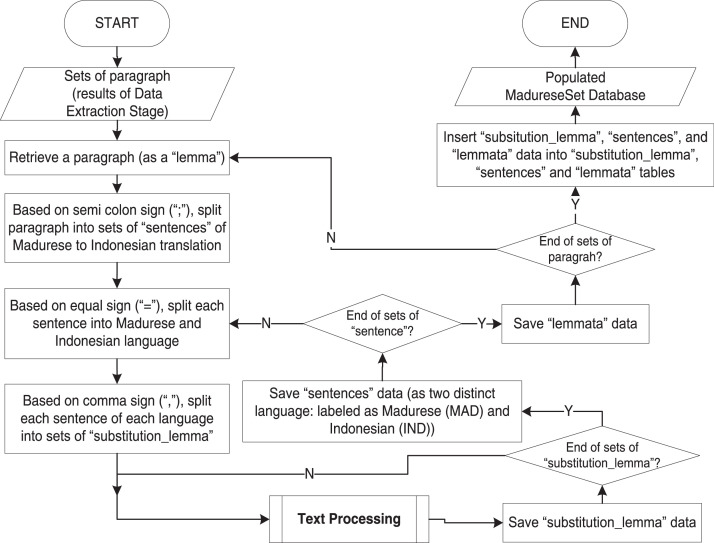
The main algorithm of madureseset data population.

**Fig. 10:**
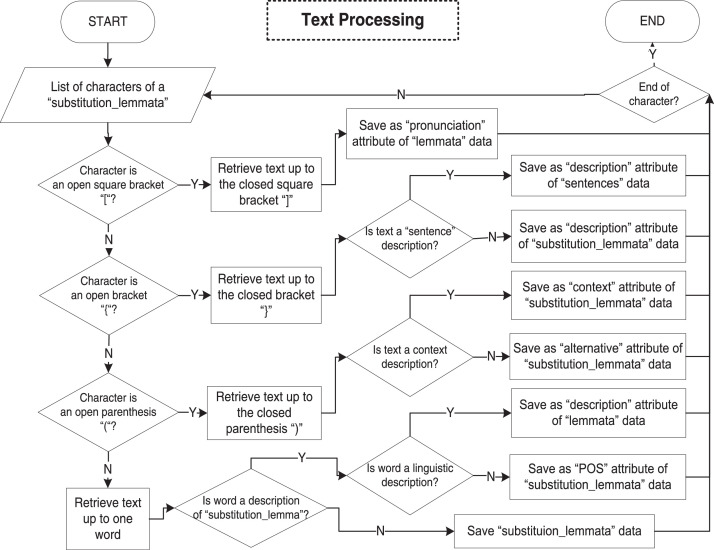
The algorithm of madureseset data population text processing procedure.

We conduct the database construction stage as two processes based on the results of the previous two stages. **First**, we construct a physical data model of database tables shown in Fig. 1. **Second**, the data population process is to automatically populate the database using a Python program based on algorithms in Figs. 9 and 10. Note that we perform manual corrections to fix errors that occurred during the automatic process due to: missing no-break space sign “ “, incomplete parentheses sign “()”, unexpected double equal sign “==” instead of single equal sign “=”, and incorrect usage of “q” character instead of “ḍ” character. In this case, we validate the database results by reviewing the number of words extracted from data in the “substitution_lemmata” table before and after the manual correction. The accuracy is calculated as the number of (correct) words before manual correction divided by the total number of words after manual correction.

Table 7 shows that the overall accuracy is 93.16%.

## Ethics Statements

The authors declare that this work does not involve human subjects, animal experiments, and data collection from social media platforms. The source of data is a public document.

**Table 7:** Validation results.

Language	Number of words extracted from data in the “substitution_lemmata” table		Accuracy
	Before Manual Correction	After Manual Correction	
Madurese	100728	105608	95.38%
Indonesian	191320	207872	92.04%
Overall	292048	313480	93.16%

## CRediT authorship contribution statement

**Noor Ifada:** Supervision, Conceptualization, Methodology, Visualization, Writing – original draft, Writing – review & editing. **Fika Hastarita Rachman:** Conceptualization, Methodology, Validation, Writing – review & editing. **M Wildan Mubarok Asy Syauqy:** Data curation, Methodology, Writing – original draft. **Sri Wahyuni:** Project administration, Resources. **Adrian Pawitra:** Validation, Resources.

## Declaration of Competing Interest

The authors declare that they have no known competing financial interests or personal relationships that could have appeared to influence the work reported in this paper.

## Data Availability

MadureseSet: Madurese-Indonesian Dataset (Original data) (Mendeley Data).
